# Prevalence of limbal stem cell deficiency at an academic referral center over a two-year period

**DOI:** 10.3389/fopht.2024.1392106

**Published:** 2024-06-24

**Authors:** Jason S. Goldberg, Daniel J. Fraser, Joshua H. Hou

**Affiliations:** ^1^ Department of Ophthalmology and Visual Neurosciences, University of Minnesota, Minneapolis, MN, United States; ^2^ Department of Ophthalmology, Hampton Veterans Affairs Medical Center, Hampton, VA, United States

**Keywords:** chemical burn, keratolimbal allograft, limbal stem cell deficiency, mucous membrane pemphigoid, Stevens-Johnson syndrome, thermal burn

## Abstract

**Aim:**

To evaluate the prevalence and clinical characteristics of limbal stem cell deficiency (LSCD) in the setting of a tertiary referral cornea practice at an academic center.

**Patient and methods:**

A retrospective chart review was performed to identify all unique medical record numbers (MRNs) presenting to a single cornea specialist (JHH) at the University of Minnesota during calendar years 2019 and 2020. Records were queried and confirmed for a diagnosis of LSCD. Clinical characteristics of identified patients, including demographics, etiology of LSCD, severity of LSCD, treatment, and best corrected visual acuity (BCVA) at final follow-up, were documented.

**Results:**

In total 1436 unique MRNs were identified over the study period. There were 61 individuals (91 eyes) diagnosed with LSCD, resulting in a prevalence of 4.25% (95% CI, 3.33-5.42). Of 91 eyes, 60 eyes were bilateral (65.9%). Among all eyes, ocular surface burns were the most common etiology (18.7%) followed by iatrogenic or medicamentosa (15.4%). There were 51 eyes (56.0%) that underwent some form of transplantation. The median BCVA at final follow-up was Snellen 20/80 (range 20/20 to no light perception).

**Conclusions:**

The prevalence of LSCD found at a cornea subspecialty tertiary referral center in our study was much higher than previously reported prevalence rates. This may reflect referral bias and potential underdiagnosis of LSCD in practices outside of subspecialty referral centers. The high prevalence rate in our study also suggests that LSCD patients are concentrated in subspecialty referral practices, with many having high morbidity disease. This constitutes a major health burden for these practices.

## 1 Introduction

Limbal stem cell deficiency (LSCD) is a devastating disease that results from excessive loss of limbal stem cells found on the ocular surface (1, 2). LSCD can occur due to chemical and thermal burns, autoimmune diseases, ocular surgeries, or other surface insults. Patients with LSCD suffer from persistent corneal epithelial defects, corneal melts, corneal scarring, and corneal conjunctivalization, which can result in significant ocular surface pain and vision loss (2). Despite being a well characterized clinical and histopathological entity with significant morbidity, LCSD has no dedicated International Classification of Diseases (ICD) code and its prevalence is heterogeneously defined. Most reported prevalence rates for LSCD are purely estimates, or extrapolated calculations based on the prevalence of co-morbid diseases such as alkaline burns, Stevens-Johnson syndrome/toxic epidermal necrolysis (SJS/TEN), or mucous membrane pemphigoid (MMP). These estimates range widely, and their accuracy is often unknown.

Orphanet estimates a disease prevalence of 1 to 5/10,000 (3), but the source for this estimate is unclear. The Holland Foundation for Sight Restoration estimates that congenital ocular surface failure causes progressive blindness in 60,000 people per year and chemical/thermal accidents impair the sight of another 100,000 each year, but the accuracy of these numbers is unknown (4). In a published interview, Dr. Virender Sangwan of the LV Prasad Eye Institute in India estimated that there are roughly 8.9 million corneally blind people in India and up to 15-20% of those patients would benefit from limbal stem cell transplant therapy (5). However, it is unclear if any of these estimates are based on formal studies. One peer-reviewed study by Bobba, et al., analyzed data from a national surveillance study in New Zealand, where they solicited voluntary responses from ophthalmologists across the country over a one-year period to determine the incidence and prevalence of LSCD. However, after 1-year, they found only 14 reported new cases of LSCD in New Zealand and authors concluded that there was likely significant underreporting and under diagnosis of LSCD in their cohort (6). This demonstrates the current difficulty with establishing accurate epidemiology numbers for LSCD.

Other published estimates of LSCD incidence and prevalence only evaluate specific subpopulations of patients with associated ocular surface diseases. These studies are all limited by the narrow patient population they examine. Ghosh, et al., found 6.86 new cases of LSCD/year with a total of 147.66 cases per year in the United Kingdom according to a prospective 6-month study (7). However, this study assessed only acute chemical injuries that presented to the emergency room. Choi, et al. found that LSCD was present in 32% of pediatric patients diagnosed with SJS/TEN; however, their study was limited by assessing only children with SJS (8).

Due to the wide range of reported rates, the true prevalence of LSCD remains unknown. This has a significant impact on our ability to understand the overall healthcare burden of LSCD. Accurate characterization of the clinical burden and/or market size of LSCD is critical for attracting research interest and potential industry investment in development of LSCD therapies. The purpose of this study was to evaluate the prevalence of LSCD in a cornea subspecialty practice and to characterize the clinical characteristics of this population of patients. This data will help improve our understanding of the unmet need and current clinical burden of LSCD.

## 2 Patients and methods

After obtaining approval from the University of Minnesota Institutional Review Board (IRB Study 0015456), electronic medical and billing records were queried for all individual patients with unique medical record numbers (MRN) seen by a single cornea specialist (JHH) during calendar years 2019 and 2020. Records were queried for billing codes ICD-9, ICD-10: H18.891, H18.892, H18.893, H18.899 (Other specified disorders of cornea [right, left, both eyes, unspecified]). These codes were used due to the electronic medical record (EMR) system (EPIC) used by the practice. The EMR uses these codes for any diagnosis or search term related to “limbal stem cell deficiency” and is the standard code consistently used by the single surgeon biller for the patient population captured in this study. Each identified patient chart was then reviewed to exclude any cases that did not have a diagnosis of LSCD. Charts for those patients meeting diagnostic criteria for LSCD were then reviewed. Age at time of diagnosis, gender, race, partial vs. total LSCD, number of quadrants involved, etiology, presence of lid abnormalities or symblepharon, laterality, treatment: medical vs. surgical, and best corrected visual acuity (BCVA) at last follow-up were extracted. The diagnostic criteria for LSCD were based on empiric findings of conjunctival epithelium growing onto the clear cornea (whorl-like keratopathy, late fluorescein staining), fibrovascular changes to the ocular surface (keratinization, corneal pannus, corneal neovascularization), and persistent epithelial defects. Our definitions for case detection adhered to LSCD diagnostic and staging criteria published by Deng et al. (2).

### 2.1 Statistical analysis

We used descriptive statistics and frequency analysis on all continuous and categorical variables among demographic and clinical data. We calculated prevalence using Taylor series linearization, and we analyzed each patient as the primary sampling unit of analysis in the prevalence calculation. This is a widely accepted method for variance calculation in large population studies (Demographic and Health Surveys, www.dhsprogram.com). For LSCD-specific analyses, each eye was the unit of analysis. Statistical significance was set at p<0.05. Statistical analyses were performed with software package STATA 17 (StataCorp, College Station, United States).

## 3 Results

### 3.1 Prevalence

Among a total of 1436 unique MRNs identified in calendar years 2019 and 2020, a total of 61 individual patients (91 eyes) were diagnosed with LSCD for a single provider in a cornea subspecialty clinic. The prevalence of LSCD among individuals was 4.25% (95% CI, 3.33-5.42). The mean age of diagnosis was 53.2 years (standard deviation 19.6, range 11-92) (**Table 1**). There were 52.5% and 47.5% identified as male and female sexes, respectively. Race data were self-reported and as follows: White (75.4%), Black (11.5%), Asian/Pacific Islander (6.6%), Middle Eastern (1.6%), Native American (1.6%), and unreported (3.3%).

**Table 1:** Demographics of patients with limbal stem cell deficiency (N = 61 individuals).

Variable	
Age at diagnosis, in years	
Mean (SD), range Median (IQR)	53.2 (19.6), 11-92 57 (21, 86)
Biological sex	
Female, n (%) Male, n (%)	29 (47.5%) 32 (52.5%)
Race	
White, n (%) Black, n (%) Asian/Pacific Islander, n (%) Middle Eastern, n (%) Native American, n (%) Unreported, n (%)	46 (75.4%) 7 (11.5%) 4 (6.6%) 1 (1.6%) 1 (1.6%) 2 (3.3%)

### 3.2 Clinical characteristics

**Figure 1:**
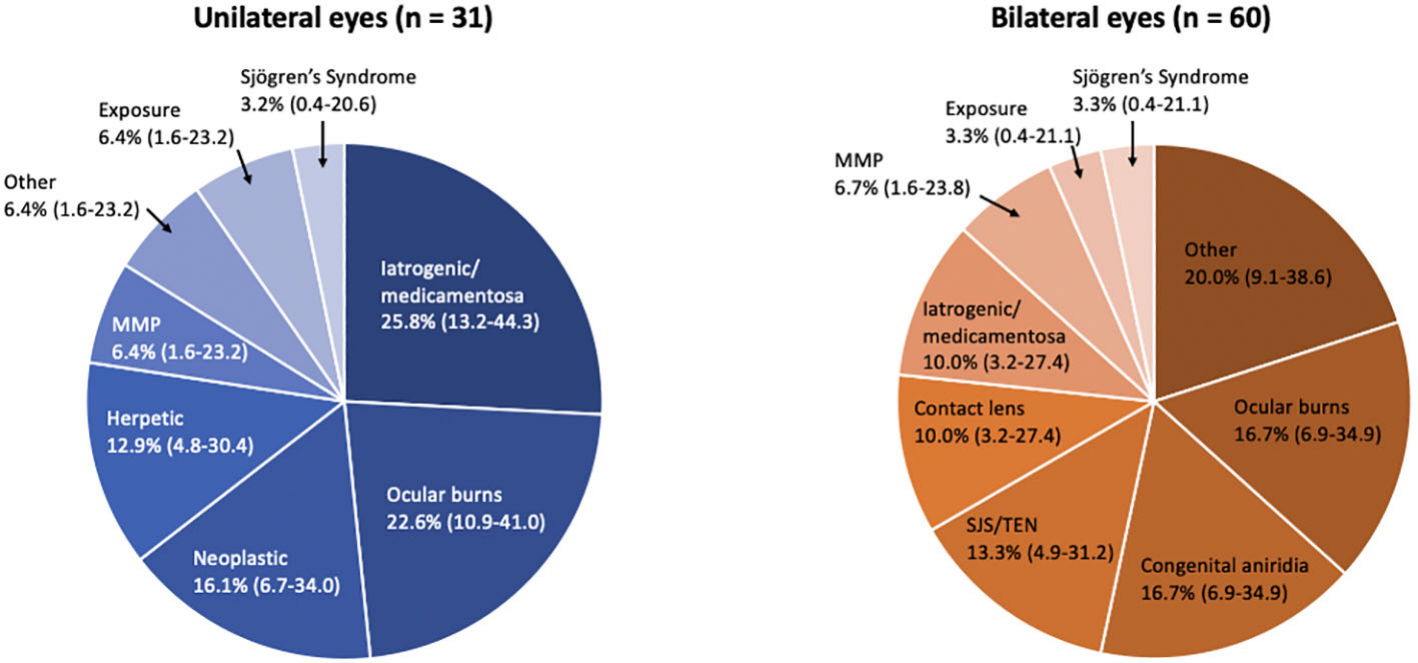
Pie chart by laterality showing etiologies of limbal stem cell deficiency (LSCD) by major categories (N = 91 eyes). Ocular burns include thermal and chemical (alkaline, acidic); Iatrogenic/medicamentosa includes sequelae of multiple ocular surgeries and any chronic topical medication therapy; Other includes Gelatinous drop-like corneal dystrophy, Graft-versus-host disease, ocular rosacea and undetermined; Neoplastic includes excision of the surface tumor and/or sequelae of mitomycin C; SJS/TEN, Stevens-Johnson syndrome/toxic epidermal necrolysis; MMP, mucous membrane pemphigoid.

Clinical characteristics are summarized in **Table 2**. Overall, 49.2% (30/61) of affected patients had bilateral disease. Of the 91 eyes with LSCD, 39.6% (36 eyes) had total LSCD. Additionally, out of 59 eyes in which the number of involved quadrants was documented in the medical records, 11.9% had LSCD involving one quadrant, 11.9% had LSCD involving two quadrants, 5.1% had LSCD involving three quadrants, and 71.2% had LSCD involving four quadrants. The primary etiologies are provided in **Table 2** and **Figure 1**. The most common causes of LSCD among all eyes were ocular surface burns (chemical and thermal, 18.7%), iatrogenic/medicamentosa (15.4%), other (15.4%), congenital aniridia (11.0%). Iatrogenic causes refer to multiple ocular surface-involving surgeries, such as trabeculectomy or glaucoma drainage device implantation. Medicamentosa refers to chronic usage of topical medications, for example, intraocular pressure lowering medications. The other category included a heterogenous mix of etiologies provided in **Table 2**. The most common etiology of unilateral LSCD was iatrogenic/medicamentosa (25.8% of unilateral cases). The most common identifiable etiology of bilateral LSCD was ocular surface burns (18.7% of bilateral cases). Overall, 42.8% of eyes had conjunctival deficiency (e.g. forniceal foreshortening, symblepharon, keratinization, or chronic conjunctival inflammation).

**Table 2:** Clinical profile, ocular comorbidities, and therapeutic management (N = 91 eyes).

	Unilateral (n = 31 eyes)	Bilateral (n = 60 eyes)
Total LSCD, n (%)	9 (29.0%)	27 (45.0%)
Partial LSCD, n (%)	22 (71.0%)	33 (55.0%)
Corneal quadrants affected, n (%)		
One Two Three Four Unable quantify	3 (9.7%) 5 (16.1%) 3 (9.7%) 11 (35.5%) 9 (29.0%)	4 (6.7%) 2 (3.3%) 0 (0.0%) 31 (50.8%) 23 (37.7%)
Global consensus staging of LSCD		
Stage 1		
A	5 (22.7%)	1 (2.7%)
B	0 (0.0%)	0 (0.0%)
C	0 (0.0%)	3 (8.1%)
Stage 2		
A	3 (13.6%)	5 (13.5%)
B	5 (22.7%)	5 (13.5%)
Stage 3	9 (29.0%)	23 (62.2%)
Etiology, n (%)		
Ocular burn Iatrogenic/medicamentosa †Other or unknown Congenital aniridia SJS/TEN Contact lens associated Mucous membrane pemphigoid Neoplastic associated Exposure keratopathy Herpetic keratitis Sjögren’s Syndrome	7 (22.5%) 8 (25.8%) 2 (6.5%) 0 (0.0%) 0 (0.0%) 0 (0.0%) 2 (6.5%) 5 (16.1%) 2 (6.5%) 4 (12.9%) 1 (3.2%)	10 (16.7%) 6 (10.0%) 12 (20.0%) 10 (16.7%) 8 (13.3%) 6 (10.0%) 4 (6.7%) 0 (0.0%) 2 (3.3%) 0 (0.0%) 2 (3.3%)
Symblepharon, n (%)	7 (22.6%)	8 (13.3%)
Ectropion or entropion, n (%)	4 (12.9%)	6 (10.0%)
Trichiasis, n (%)	9 (29.0%)	10 (16.7%)
Transplantation, n (%)		
Keratolimbal allograft Autologous SLET Penetrating keratoplasty Keratoprosthesis Deep anterior lamellar keratoplasty	9 (29.0%) 2 (6.5%) 3 (9.7%) 1 (3.2%) 1 (3.2%)	26 (42.6%) 0 (0.0%) 6 (10.0%) 3 (4.9%) 0 (0.0%)
Conservative management, n (%)	15 (48.4%)	25 (41.7%)

SJS/TEN, Stevens-Johnson syndrome/toxic epidermal necrolysis; SLET, simple limbal epithelial transplantation.

†Other etiologies: unknown (5 eyes), Gelatinous drop-like corneal dystrophy (2 eyes), severe infectious keratitis ulcers (2 eyes), ocular rosacea (2 eyes), sickle cell/thalassemia associated (2 eyes), graft-versus-host disease (1 eye).

### 3.3 Management

There were 51 eyes (56.0%) that underwent some form of transplantation, mostly keratolimbal allografts to address LSCD (38.4%, 35 of 91). Four keratoprosthesis were performed (4.4%) given inability for the patients to be on long-term immunosuppression. There were 9 eyes (9.9%) that underwent PKP and one eye that underwent a deep anterior lamellar keratoplasty (DALK). All keratoplasties were done in a staged manner following limbal stem cell transplantation. The 40 eyes (44.0%) managed conservatively were treated using frequent preservative free artificial tears, bandage contact lenses, scleral lenses, serum tears, topical corticosteroids, cenegermin, topical tacrolimus or cyclosporine, and/or surgical procedures to improve ocular surface coverage, such as tarsorrhaphy or (in severe cases) Gunderson conjunctival flaps (3 eyes).

### 3.4 Visual outcomes

**Figure 2:**
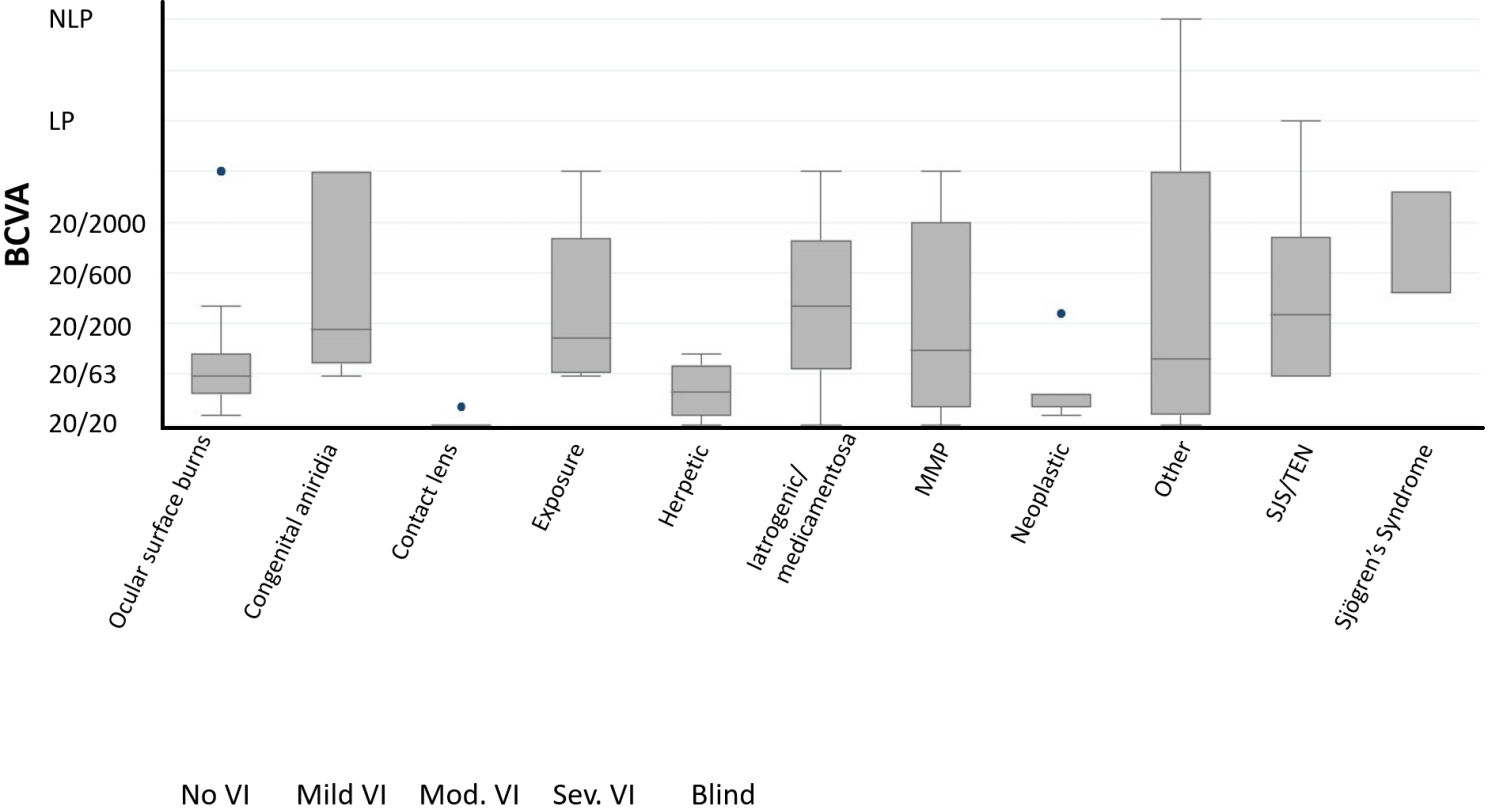
Best-corrected visual acuity (BCVA) at final follow-up by major categories of etiologies (N = 91 eyes). NLP, No light perception; LP, light perception.

**Figure 3:**
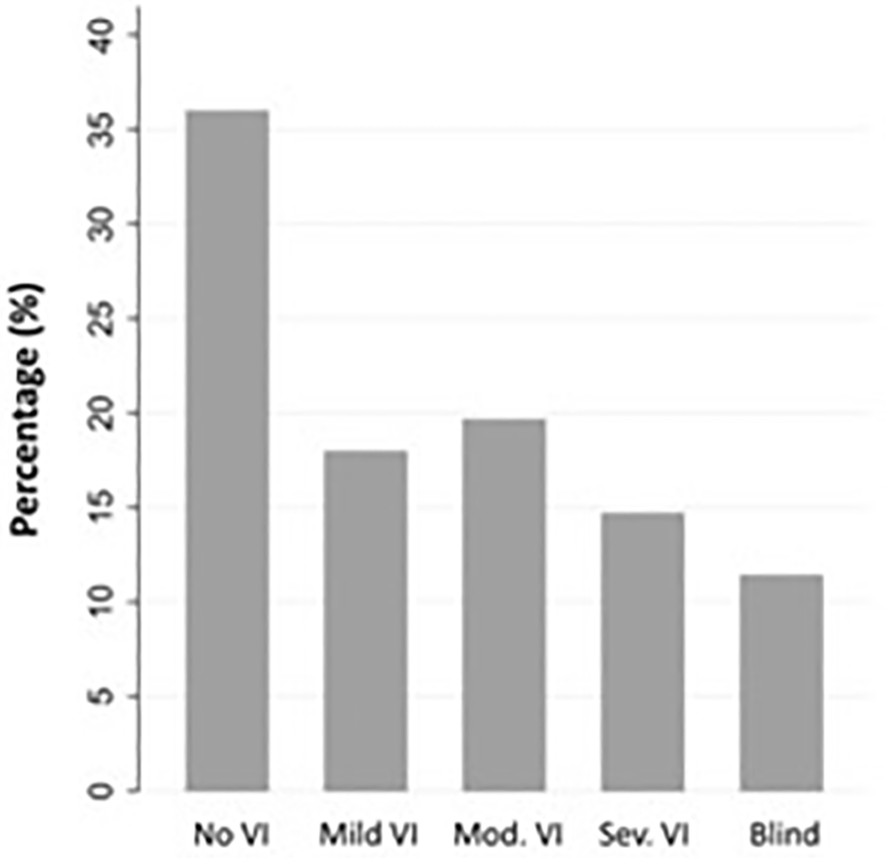
Best-corrected visual acuity (BCVA) at final follow-up by International Classification of Diseases (ICD) 11 distance visual impairment categories (N = 61 individuals). No VI: no visual impairment (Snellen 20/40 or better), Mild VI: mild visual impairment (Snellen 20/50 and 20/60), Mod VI: moderate visual impairment (worse than Snellen 20/60 to 20/200), Sev VI: severe visual impairment (worse than 20/200 to 20/400), Blind (worse than Snellen 20/400).

Among 91 affected eyes, the median BCVA at final follow-up was Snellen 20/80 (range 20/20 to no light perception). By etiologies, LSCD associated with contact lens overwear had the best median BCVA of 20/20, followed by neoplastic and herpetic keratitis both with median BCVA of 20/40 (p<0.001, each etiology in comparison to all others, Multivariate analysis of variance and covariance [MANOVA] test) (**Figure 2**). The proportion of individuals with blindness (BCVA in the better seeing eye worse than 20/400 as defined by the World Health Organization/ICD blindness) was 11.5% (**Figure 3**).

## 4 Discussions

This study provides important data to help establish the overall healthcare burden of LSCD. Based on the clinical characteristics of patients in this cohort, LSCD is a high morbidity disease with 11.5% of patients having BCVA 20/400 or worse in the better seeing eye at final follow-up and 56.0% of eyes requiring surgical therapy. Though rare, the prevalence of LSCD in our study cohort was dramatically higher than the overall U.S. prevalence reported by Orphanet (4.25% vs. 0.0001-0.0005%). This suggests that LSCD patients are heavily concentrated in cornea subspecialty and tertiary referral practices. Ophthalmologists outside of these practices may rarely encounter this disease and as a result may lack familiarity with making the diagnosis. This may contribute to significant underdiagnosis of the disease. The lack of a specific ICD-10/-11 code for LSCD may further exacerbate this problem.

Overall, ocular surface burns were the most common cause of LSCD in our cohort, accounting for 18.7% of cases. This was consistent with other published studies that found high rates of ocular surface burns among LSCD patients (9). In a study from India reviewing clinical characteristics of LSCD patients at two major referral centers, ocular surface burns accounted for 84% of all unilateral LSCD cases and 30% of all bilateral LSCD cases. The higher percentage of patients presenting with ocular surface burns in their cohort compared to ours may be due to the higher rates of burn trauma overall in their India-based centers. Of note, congenital aniridia was a significant proportion of bilateral cases in both our study and the study from India (16.7% of bilateral cases in our study versus 9% bilateral cases in Vazirani, et al), demonstrating the significance of the disease globally. Furthermore, in a study done by Cheung et al., in 2021, the rate of patients presenting with bilateral LSCD was as high as 70%. The common etiologies for presentation were similar to this study e.g. congenital aniridia, chemical or thermal injuries, contact lens, Stevens-Johnson syndrome, and iatrogenic causes. However, a big difference was that the rate of congenital aniridia was significantly higher in this study at around 30%. Also, iatrogenic causes were only 7% of the data, which shows how greatly the causes for this condition can vary from study to study (10).

Medicamentosa and iatrogenic causes contributed to 15.4% of the LSCD diagnoses in these patients. According to a study done at the University of Minnesota examining iatrogenic LSCD from 1986-1996, traumatic disruption of the stem cells in surgery is hypothesized to be the primary causative mechanism for development of iatrogenic LSCD. Such trauma is believed to increase the susceptibility of stem cells to external disease and toxic influences, thus resulting in development of LSCD (11, 12).

In terms of visual outcomes, the contact lens-associated cohort had the best visual acuity at final assessment. This is similar to findings from a 2015 study from the Cincinnati Eye Institute. In that study, patients with contact-lens related LSCD were examined and followed long-term for treatment outcomes. Their study showed that the contact-lens related LSCD showed great response and improvement to limbal stem cell transplant. In all but 1 patient, BCVA improved to at least 20/30 from 20/70 or worse. The agreement between our study and theirs supports the conclusion that contact-lens related LSCD patients have a better prognosis than other etiologies of LSCD (13–15).

In our study, the rate of bilateral disease in affected patients was found to be 49.2%. This is close to the rate found by Vazirani et al, in which 40.6% (540/1331) of patients had bilateral disease (9). The small difference in rates may be due to the difference between the patient populations in US and India (higher rates of unilateral burns in India), or differences in diagnostic criteria for LSCD used in each study. Though our study had a higher rate of bilateral disease, only 39.6% (36/91) of eyes in our study had total LSCD compared to 68.4% (1239/1812) of eyes in their study (9). This suggests overall disease severity may have been higher in their India-based cohort.

The management of LSCD is challenging as the benefits of treatment must be balanced against the inherent risks. Limbal stem cell transplantation is the recognized treatment for severe LSCD (stage IIB, III) (16); however, limbal stem cell transplants are associated with significant rates of rejection. Post-operative use of systemic immunosuppression is critical for achieving optimal outcomes, but numerous side effects are associated with their use. Because of this, surgical intervention is usually reserved for cases refractory to medical therapy, such as aggressive lubrication, topical anti-inflammatory medications, bandage contact lenses, scleral lenses, and amniotic membranes (17–22). Failing that, patients often progress to surgical intervention for advanced or refractory disease (17). These therapies can include boston keratoprosthesis, and limbal stem cell transplant. In our study, over half (56%) of the LSCD cohort required surgical management for their condition. According to Iyer et al, 2020, this split in treatment is typical for management of this condition.

Both the study by Iyer et al, and the global consensus by the LSCD Working Group of the Cornea Society (23) support our findings of a near 50/50 split in the management of this condition. In addition to this, the surgical techniques used to manage LSCD have varying outcomes and can depend on the severity and laterality of the disease. Both keratoprosthesis and limbal stem cell transplantation have been used in the management of severe LSCD with improved outcomes over the past two decades (16, 24–27). These improvements and successes in managing bilateral LSCD have been demonstrated in multiple case studies such as the one by Vazirani et al. (2016), and other examples (28–32). Furthermore, the global consensus on the management of LSCD by Deng et al., 2020, recommends a combination of surgical and medical treatments for this condition, which again is similar to our study (23, 33–35).

Some weaknesses of this study are that it was a retrospective study and limited to a single cornea subspecialty clinic. The difficulty in tracking patients with a diagnosis of LSCD due to the lack of specific ICD-10 codes and the fact that these patients all had code-able co-morbid diseases, also meant that cases of LSCD could have been missed. Because LSCD is a clinical diagnosis made at the slit lamp with no definitive diagnostic testing, significant experience is needed to consistently diagnose LSCD. By focusing our study on a single provider skilled in the diagnosis of LSCD, our study was able to provide an upper limit estimate of LSCD prevalence. On the other hand, the inherent referral bias from analyzing only a single referral practice means that the prevalence of LSCD in our study cannot be simply extrapolated to the wider U.S. patient population. The major limitation of this study is the restriction of scope to one provider at a single referral center, which may result in an overestimation of the prevalence, thus creating the likely upper limit for the prevalence of this condition. Further research across multiple sites and over a longer period will help fine tune this prevalence. However, our study does suggest that LSCD represents a significant disease burden in corneal subspecialty practices where LSCD patients are likely to be concentrated.

## 5 Conclusions

Overall, LSCD is a high morbidity disease with a significant prevalence in subspecialty and referral practices. The highly non-uniform concentration of LSCD patients in specialty and referral practices should be carefully considered in future epidemiology studies of LSCD. Future directions for this study should include expanding the data collection to include multiple centers and potentially prospectively standardizing the criteria for inclusion, diagnostic testing, and codified clinical documentation. Accurate estimates of LSCD prevalence and market size remain critical for drawing industry and academic interest in therapeutic developments for this disease.

## Data availability statement

The raw data supporting the conclusions of this article will be made available by the authors, without undue reservation.

## Author contributions

JG: Writing – original draft, Writing – review & editing, Conceptualization, Data curation, Investigation, Methodology, Project administration, Resources, Supervision, Formal analysis, Validation, Visualization. DF: Writing – original draft, Writing – review & editing, Data curation, Investigation. JH: Writing – original draft, Writing – review & editing, Conceptualization, Data curation, Formal analysis, Funding acquisition, Investigation, Methodology, Project administration, Resources, Software, Supervision, Validation, Visualization.
